# Neutrophil extracellular trap inhibition increases inflammation, bacteraemia and mortality in murine necrotizing enterocolitis

**DOI:** 10.1111/jcmm.15338

**Published:** 2020-06-08

**Authors:** Hala Chaaban, Kathryn Burge, Jeffrey Eckert, Ravi S. Keshari, Robert Silasi, Cristina Lupu, Barbara Warner, Marilyn Escobedo, Michael Caplan, Florea Lupu

**Affiliations:** ^1^ Department of Pediatrics, Division of Neonatology University of Oklahoma Health Sciences Center Oklahoma City OK USA; ^2^ Oklahoma Medical Research Foundation, Cardiovascular Biology Research Program Oklahoma City OK USA; ^3^ Department of Pediatrics, Division of Newborn Medicine Washington University School of Medicine St. Louis MO USA; ^4^ University of Chicago Pritzker School of Medicine Chicago IL USA

**Keywords:** necrotizing enterocolitis, neutrophil extracellular traps, nucleosomes

## Abstract

Necrotizing enterocolitis (NEC) is a devastating gastrointestinal disease affecting primarily premature infants. The disease is characterized by intestinal inflammation and leucocyte infiltration, often progressing to necrosis, perforation, systemic inflammatory response and death. Neutrophil extracellular traps (NETs), denoting nuclear DNA, histone and antimicrobial protein release, have been suggested to play a role in NEC. This study aimed to determine the role of NETs in NEC and explore the effect of chloramidine, a NET inhibitor, on a murine NEC‐like intestinal injury model. Blood and intestinal tissues were collected from infants diagnosed with ≥ Stage II NEC, and levels of nucleosomes and NETs, respectively, were compared with those of case‐matched controls. In mice, NEC was induced with dithizone/*Klebsiella*, and mice in the treatment group received 40 mg/kg chloramidine. Bacterial load, intestinal histology, plasma myeloperoxidase and cytokine levels, and immunofluorescent staining were compared with controls. Nucleosomes were significantly elevated in both human and mouse NEC plasma, whereas NET staining was only present in NEC tissue in both species. Chloramidine treatment increased systemic inflammation, bacterial load, organ injury and mortality in murine NEC. Taken together, our findings suggest that NETs are critical in the innate immune defence during NEC in preventing systemic bacteraemia.

## 1 INTRODUCTION

Necrotizing enterocolitis (NEC) is the most common gastrointestinal emergency and the leading surgical cause of death in the neonatal intensive care unit.^1^ It is characterized by inflammation and patchy necrosis of the bowel wall that can rapidly progress to sepsis. Despite advances in clinical care, prevalence and mortality from NEC have not changed. Approximately 7%‐10% of premature infants develop NEC and 20%‐30% of those do not survive. Mortality is even higher, and up to 50% in the very‐low‐birthweight infants (<1000 g).^1^, ^2^, ^3^

Whereas the aetiology of NEC is still unclear, evidence suggests that prematurity, formula feeding and an altered intestinal microbiome play central roles in the pathogenesis of the disease.^4^ Histologically, NEC is characterized by inflammation, massive leucocyte infiltration, epithelial necrosis and bacterial translocation.^5^, ^6^ Although neutrophil infiltration has been long recognized, the beneficial or detrimental contributions of these cells in NEC remain controversial.^5^, ^7^ Neutrophils are first‐line responders eradicating most infectious organisms by a combination of phagocytosis, degranulation or the relatively novel mechanism of neutrophil extracellular traps (NETs).^8^ In NEC, neutrophils appear necessary for pathogen clearance, as the lack of, or dysfunctional neutrophils aggravate intestinal mucosal injury in a murine model of NEC. However, excessive neutrophil recruitment or inadequate neutrophil clearance could cause collateral damage through the release of toxic products and reactive oxygen species.^7^, ^9^

The potential role of NETs in the pathogenesis of NEC is a relatively new area of investigation.^10^, ^11^, ^12^ NETs are large web‐like structures formed by neutrophil nuclear DNA enriched with histones as well as cytoplasmic and granular antimicrobial proteins, such as neutrophil elastase (NE) and myeloperoxidase (MPO).^13^ These physical entrapments are either released in a rapid non‐lethal process, or more commonly hours after stimulation, resulting in a cell death process distinct from apoptosis or necrosis.^13^ A key step in NET formation is the citrullination of histones H2B, H3 and H4 via the enzyme peptidyl arginine deiminase 4 (PAD4).^14^ NETs are cleared in the body through degradation by plasma deoxyribonuclease 1 (DNAse 1), DNase1L3 and DNAse III, and subsequent macrophage phagocytosis.^15^ Whereas NETs are known to capture and destroy offending microorganisms, excessive NET formation or delayed clearance of NETs or NET components specifically histones contributes to pathological conditions like sepsis, thrombosis and transfusion‐associated acute lung injury.^16^, ^17^, ^18^, ^19^

In human neonates, neutrophils display an intrinsic delay in NET formation, yet these immune cells are still capable of releasing functionally competent NETs.^16^, ^20^ NETs were identified in a lipopolysaccharide (LPS)/hypoxia/formula‐feeding NEC model, where NET inhibition resulted in less tissue damage and inflammation, and reduced mortality.^10^ Notably, in premature infants with NEC, it is postulated that an altered intestinal microbiome and impaired epithelial barrier are associated with developing the disease.^21^ Whether NETs offer protection against abnormal intestinal pathogens or contribute to pathology in models of NEC that require intestinal infection is still unclear.

The aims of the study were to confirm the presence of NETs and circulating nucleosomes (histone octamers bound to DNA and released during cell damage and death) in human NEC, and examine the effect NET inhibition using chloramidine (Cl‐amidine), a pan‐PAD and NET inhibitor,^22^ on the incidence and severity of NEC in a mouse model of dithizone/*Klebsiella* infection.

## 2 METHODS

### 2.1 Study design

This observational study was approved by the Institutional Review Board (IRB, protocol # 2472) the University of Oklahoma Health Sciences Center (OUHSC). Informed consent was obtained from all parents or legal guardians of neonates involved in this study. Serum was collected from premature human infants prospectively at the time of NEC diagnosis. NEC diagnosis was based on clinical manifestations and radiographic findings established by the modified Bell's staging criteria.^23^ Accordingly, radiographic finding of pneumatosis intestinalis with significant intestinal dilation and ileus with or without portal vein gas or ascites was defined as NEC, Stage II. Additional ascites and/or bowel perforation defined advanced NEC, Stage III. Only infants with Stage II or III NEC were included in the NEC group. Controls were matched two‐to‐one to NEC cases for gestational age (GA) at birth (±1 week), birthweight (±200 g) and post‐natal age (±1 week). Human ileal tissue samples were acquired from a repository at the OUHSC.

### 2.2 Human nucleosome analysis

Circulating nucleosomes, a marker for NET release,^24^, ^25^ were measured from human serum using ELISA (Cell Death Detection ELISA^plus^ kit, Version 14: Roche Diagnostics, Indianapolis, IN), per manufacturer's instructions. Briefly, the ELISA utilized 20 μL of plasma and 80 μL of immunoreagent per well, and samples were run in duplicate. Samples were measured in relation to background and positive control.

### 2.3 Immunofluorescent staining of human ileal NEC tissues

Human ileal tissue samples, stored as paraffin blocks, were cut to 5‐µm sections and mounted on charged slides. Following deparaffinization, sections were rehydrated utilizing serial dilutions of water and ethanol. Antigen retrieval was accomplished using 10 mmol/L sodium citrate buffer, pH 6.0. Sections were incubated with rabbit anti‐H4 Cit3 IgG1 (citrullinated histone H4; EMD Millipore, Burlington, MA), followed by Cy3 conjugated donkey anti‐rabbit IgG (Jackson ImmunoResearch, West Grove, PA). After blocking with unconjugated donkey anti‐rabbit IgG Fab fragments (Jackson ImmunoResearch), sections were incubated with rabbit anti‐neutrophil elastase IgG (Calbiochem, San Diego, CA), followed by FITC‐conjugated donkey anti‐rabbit IgG (Jackson ImmunoResearch) and mounted using Vectashield Antifade Mounting Medium (Vector Laboratories, Burlingame, CA) containing DAPI as a nuclear stain. Isotype‐matched controls were used as negative controls. Sections were visualized with a Nikon C1 confocal microscope (Nikon, Melville, NY).

### 2.4 Mouse model of NEC

All animal experiments were approved by the Institutional Animal Care and Use Committee (IACUC) of the University of Oklahoma Health Sciences Center and performed according to recommendations in the Guide for the Care and Use of Laboratory Animals. Time‐pregnant CD‐1 mice were purchased from Charles River Laboratory, Wilmington, MA, and were housed under standard conditions with free access to standard laboratory feed and water. Mice pups were delivered naturally and were dam‐fed before experiments. On P15, the day of experimentation, pups were separated from their mothers and maintained in a temperature‐ and humidity‐controlled chamber. For the NEC challenge in the study, we used a modified Paneth cell ablation and *Klebsiella* infection model, also known as the dithizone/*Klebsiella* (DK) model.^26^ Briefly, CD‐1 mouse pups of both sexes were divided into three groups: (a) sham (n = 10), (b) NEC (n = 20) and (c) NEC + Cl‐amidine (n = 20). Pups in the NEC groups received an intraperitoneal injection of dithizone (33 mg/kg bodyweight; Sigma‐Aldrich, St. Louis, MO) dissolved in 25 mM lithium carbonate solution or an equivalent volume of vehicle alone (Supplemental Figure 1). At six hours post‐injection, pups received an enteral gavage of 1 × 10^8^ CFU (colony‐forming units)/gram bodyweight of *Klebsiella pneumoniae* (ATCC#10031, Manassas, VA). Pups in the Cl‐amidine group received 40 mg/kg bodyweight intraperitoneal administration of the NET inhibitor Cl‐amidine (Calbiochem Cat. 506282, Millipore, Burlington, MA) in phosphate‐buffered saline (PBS) both 30 minutes before the bacterial challenge and 3 hours post‐challenge. Cl‐amidine dosing was determined considering prior studies of NET inhibition in sepsis^27^ and dextran sulphate sodium colitis.^28^ Pups were continuously monitored for distress and survival for 10 hours, after which surviving pups were killed, and blood and tissues were collected.

### 2.5 Blood culture

In a separate set of experiments, the DK challenge was conducted as described above, and blood was collected from all pups by cardiac puncture 1, 3, 6 or 10 hours after bacteria administration (n ≥ 6 per group). Serial dilutions of the blood using sterile PBS were plated in triplicate onto square gridded agar plates (D210‐16, Simport, Beloeil, Quebec, Canada) using the track dilution method. Plates were read the following morning after incubating aerobically overnight at 37°C and used to assess levels of bacteraemia.

### 2.6 Histological evaluation and immunofluorescent staining of mouse NEC ileum

Mouse terminal ileal tissues were fixed with 10% formalin buffer, paraffin‐embedded and stained with haematoxylin and eosin for microscopic examination. Mucosal intestinal injury was evaluated by two blinded pathologists and graded on a 4‐point scale developed by Jilling et al^29^: grade 0, no injury (normal); grade 1, sloughing of villous tip cells; grade 2, mid‐villous damage; grade 3, denudation of epithelium with loss of villi, crypts intact; grade 4, transmural necrosis. Histological analysis was performed on 3‐5 sections per specimen, and the highest score per specimen was recorded. Histological scores ≥ 2 were defined as NEC. Immunofluorescent staining of mouse NEC tissue largely followed the procedures outlined above for human tissue samples. After deparaffinization, rehydration and antigen retrieval, sections were incubated with rabbit anti‐MPO IgG (Epitomics, Burlingame, CA), followed by Cy3‐conjugated donkey anti‐rabbit IgG (Jackson ImmunoResearch, West Grove, PA). Additionally, after blocking with unconjugated donkey anti‐rabbit IgG Fab fragments (Jackson ImmunoResearch), sections were incubated with rabbit anti‐MPO (EMD Millipore), followed by FITC‐conjugated donkey anti‐rabbit IgG (Jackson ImmunoResearch) and mounted using Vectashield Antifade Mounting Medium (Vector Laboratories, Burlingame, CA) containing DAPI as a nuclear counterstain. Quantification of fluorescence intensity was obtained using the ImageJ program (National Institutes of Health, Bethesda, MA). The threshold for each stain was adjusted to the same level for all samples.

### 2.7 Circulating nucleosome, MPO and cytokine analysis

Mouse circulating nucleosomes were measured as described above. MPO activity in mouse plasma was measured using a Fluoro MPO^TM^ kit (Cell Technology, Mountain View, CA). Fluorescence excitation was measured at 570 nm, and emission was measured at 600 nm. Samples were quantified via comparison to the standard curve of known values.

Mouse serum cytokines such as TNF‐α, growth‐regulated oncogene‐alpha (GRO‐α), interleukin 6 (IL‐6), IL‐1β, IL‐10 and monocyte chemoattractant protein 1 (MCP‐1) were measured using immunofluorescence technology (xMAP 6‐plex cytokine/chemokine magnetic bead panel, EMD Millipore, Burlington, MA) as per manufacturer's instructions. Samples were run on a BioPlex 200 instrument (Bio‐Rad, Hercules, CA) in duplicate, and cytokine levels were quantified via comparison to a 4PL algorithm standard curve.

### 2.8 Complete blood counts (CBCs)

Mouse blood was collected via cardiac puncture into EDTA‐coated capillary tubes. Cell counts were analysed using a Hemavet 950FS Veterinary Multi‐species Hematology System (Drew Scientific, Miami Lakes, FL).

### 2.9 Statistical analysis

All statistical analyses were performed using GraphPad Prism 6 (version 6.07) for Windows (GraphPad Software, La Jolla, CA, www.graphpad.com). Data are presented as mean ± SEM, and differences between and among groups were analysed using either unpaired *t* test or one‐way ANOVA followed by Tukey test where appropriate. Survival data were analysed using Kaplan–Meier curves (Mantel–Cox logrank test). Data were considered significant when *P* < 0.05.

## 3 RESULTS

### 3.1 Circulating nucleosomes are present in premature human infants with NEC

**FIGURE 1:**
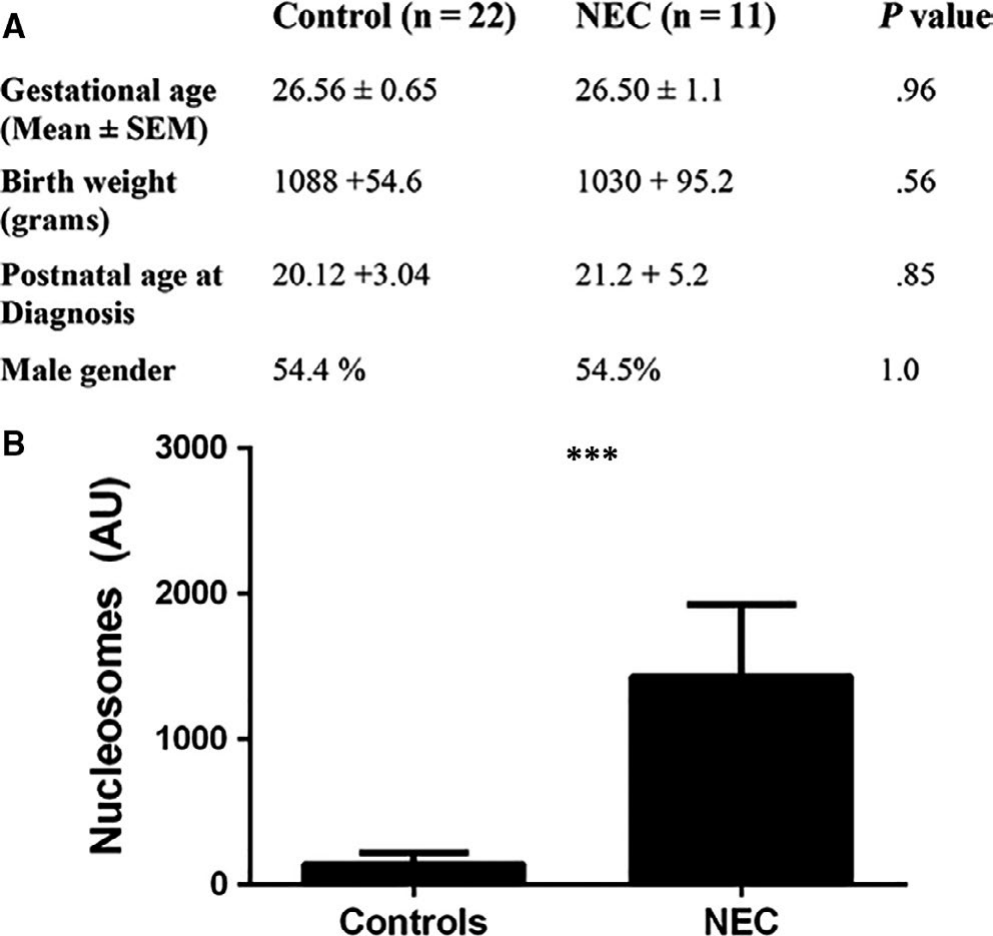
A, Demographic data from preterm infants who developed NEC stage > II according to modified Bell's criteria, compared with control. B, Circulating nucleosome levels in human premature NEC cases compared with controls. Values denote mean ± SEM by unpaired *t* test. ***P < 0.01. NEC, necrotizing enterocolitis

To determine whether nucleosomes are released during NEC in premature infants, we measured circulating levels of histone‐DNA complexes in infants diagnosed with NEC. First, we compared the demographic characteristics between cases and controls. As shown in Figure 1A, there were no differences in GA, weight or gender between NEC cases and controls. Levels of circulating nucleosomes were significantly higher in NEC cases compared with controls (Figure 1B; *P* = 0.0012). Though not statistically significant, nucleosomes levels were higher in infants with the more severe NEC stage III than II (Supplemental Figure 2).

### 3.2 Presence of neutrophil extracellular traps in human NEC ileum

**FIGURE 2:**
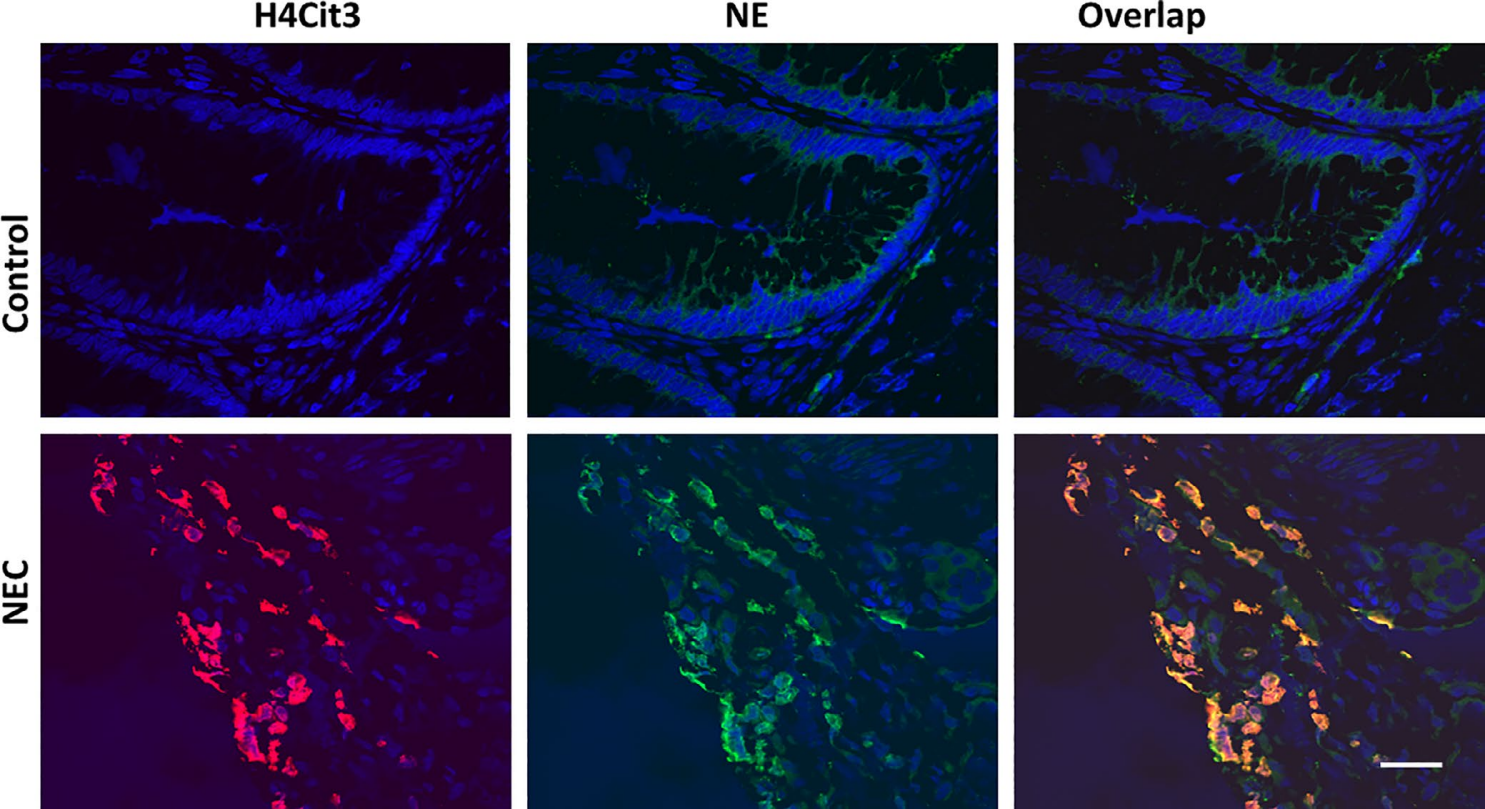
Double immunofluorescence and confocal microscopy of human premature NEC ileum with anti‐citrullinated histone H4 Cit3 (H4Cit3; Cy3, red) and anti‐neutrophil elastase (NE; FITC, green) IgGs. Blue, DAPI for nuclear staining. Bar, 50 µm

Given the elevated levels of circulating nucleosomes present in human preterm NEC, we sought to determine whether NET formation occurs in the ileum of preterm neonates with NEC. We performed immunofluorescent staining on ileal samples from infants with severe NEC versus controls (healthy margins of small intestine from infants less than one‐month‐old undergoing surgery for intestinal atresia). NET formation was identified by the co‐localization of NE, a marker for neutrophil activation^30^ and H4Cit3 as a marker for NET release.^31^, ^32^ Figure 2 illustrates abundant NET formation in NEC samples. No H4Cit3 or NE staining was detected in sections from controls.

### 3.3 NET inhibition in DK NEC model is associated with increased mortality and bacteraemia

NEC was induced using a modified dithizone/Klebsiella (DK) NEC‐like model. This model is characterized by an intestinal dysbiosis phenotype that is similar to that of human infants who develop NEC.^33^ Intraperitoneal (i.p.) treatment with the NET inhibitor, Cl‐amidine (40 mg/kg), was given at 30 minutes before, and 3 hours after, bacterial gavage. Animals were monitored for a total of 10‐hours post‐bacterial gavage for signs of distress and survival.

**FIGURE 3:**
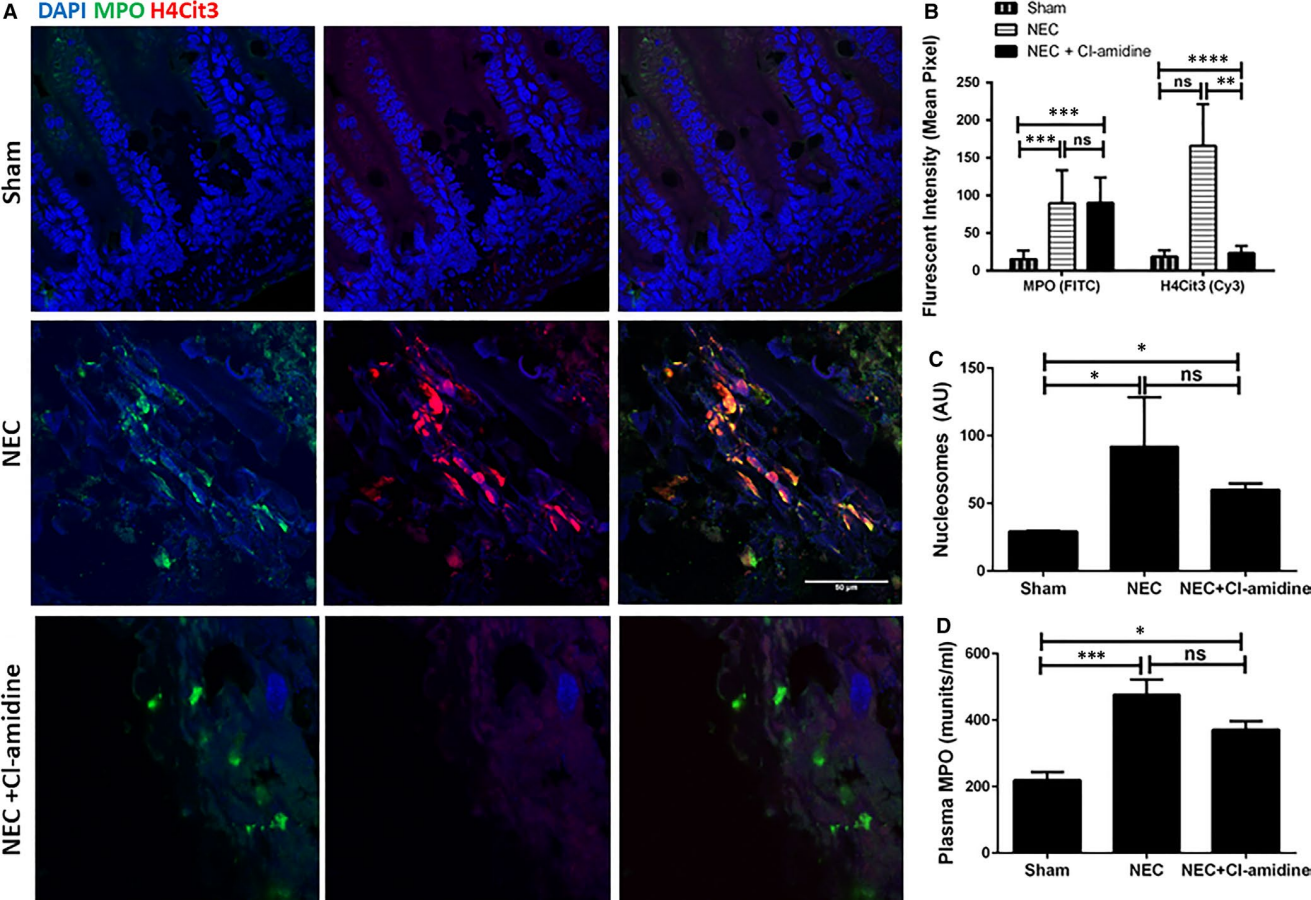
A, Double immunofluorescence and confocal microscopy of NEC ileum from mouse pups with anti‐citrullinated histone H4 Cit3 (H4Cit3; Cy3, red) and anti‐myeloperoxidase (MPO; FITC, green) IgGs. Blue, DAPI for nuclear staining. Bar, 50 µm. B, Fluorescence intensity for FITC (MPO) and Cy3 (H4Cit3) was measured for each group using ImageJ software (NIH—Bethesda, USA). **P < 0.01, **P < 0.001, ***P < 0.0001. C, Plasma nucleosome levels in mouse shams, NEC and NEC + Cl‐amidine pups after 1 hour post‐gavage. *P < 0.05. D, Plasma MPO levels in mouse NEC, NEC + Cl‐amidine and sham pups. ***P < 0.001, ns, *P* > .05. Values denote mean ± SEM by one‐way ANOVA. AU, absorbance units; NEC, necrotizing enterocolitis

Double immunofluorescence staining performed on mouse ileal samples identified NET formation by co‐localizing MPO and H4Cit3. NET formation was abundant in the small intestine of pups in the NEC group. As expected, no H4Cit3 or MPO staining was detected in sections from shams, whereas pups in the NEC + Cl‐amidine group had only MPO‐positive cells with no evidence of H4Cit3 staining (Figure 3A,B). Next, we determined the effect of Cl‐amidine on circulating nucleosomes and found significantly elevated levels in NEC pups versus sham (*P* = 0.0455); however, nucleosome levels in NEC + Cl‐amidine pups did not differ significantly from NEC pups (Figure 3C), indicating that circulating nucleosomes in NEC originates not only from NETs but also from tissue injury. Plasma MPO levels were also significantly higher in both NEC groups compared with shams: *P* = 0.0004 for NEC, and *P* = 0.0249 for NEC + Cl‐amidine. There was no significant reduction in MPO levels in NEC + Cl‐amidine pups compared with NEC pups alone (Figure 3D; *P* = 0.0781).

**FIGURE 4:**
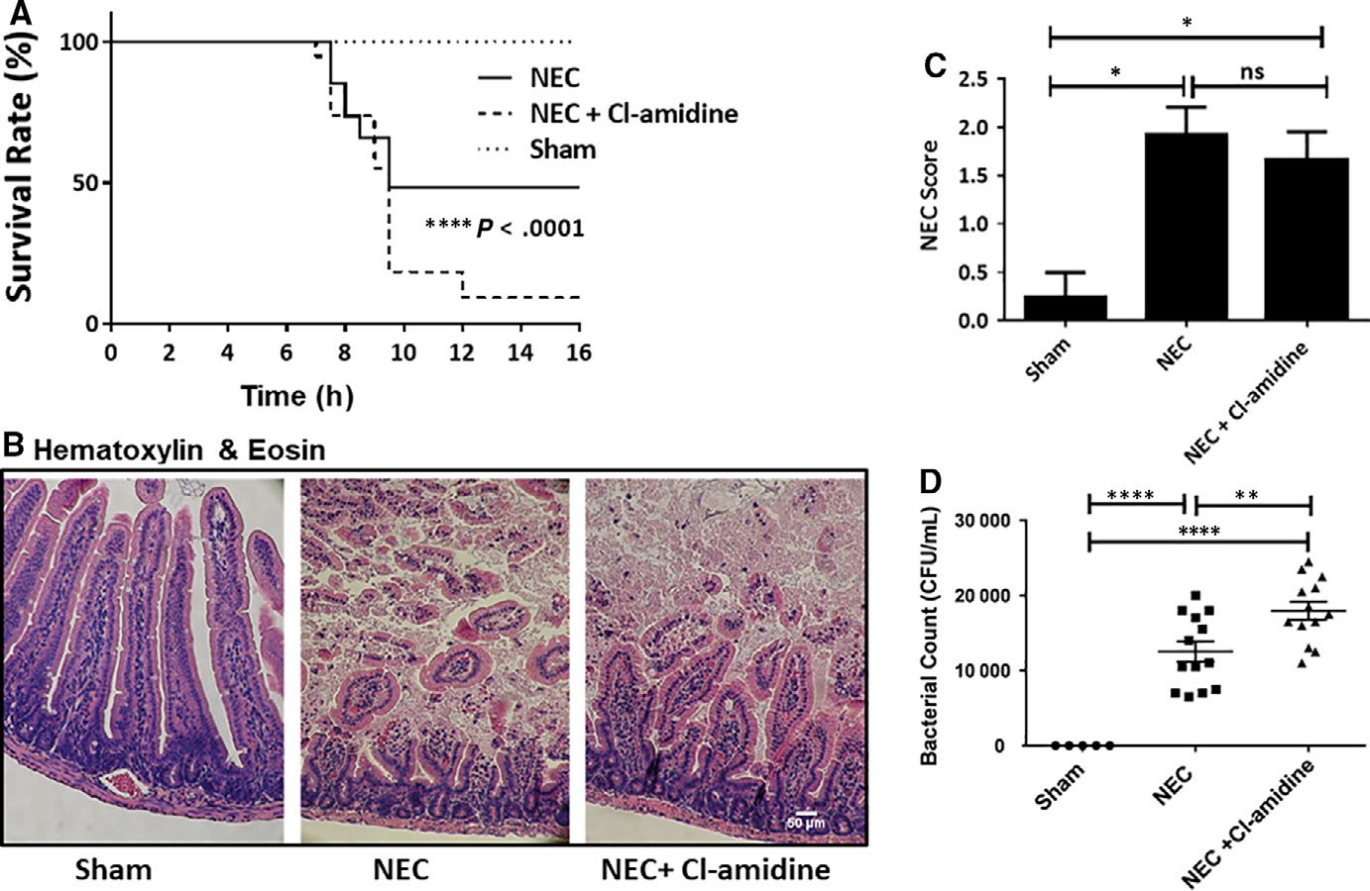
A, Cl‐amidine‐mediated inhibition of NET production during murine NEC drastically reduces pup survival. ****P < 0.0001. B, Representative haematoxylin and eosin staining in the ileum of mouse pups from sham, NEC and NEC + Cl‐amidine groups. C, Histological intestinal injury scores for pups undergoing NEC. *P < 0.05. Bar, 50 µm. D, Bacterial counts in mouse pups 1 hour post‐gavage. **P < 0.01. Values denote mean ± SEM by Student's *t* test or one‐way ANOVA with Turkey's multi‐comparison test. CFU, colony‐forming units; NEC, necrotizing enterocolitis

Surprisingly, Cl‐amidine treatment almost doubled the mortality in pups exposed to DK‐induced NEC, as shown by the Kaplan–Meier survival curve (Figure 4A): 94.7% mortality in NEC + Cl‐amidine group compared with 47.6% mortality in NEC alone (*P* < 0.0001). All shams survived until the experimental endpoint, showing no signs of NEC. Furthermore, increased pup mortality in the NEC + Cl‐amidine group does not correlate with increased intestinal injury, as the incidence (76.9% vs 77.8%, NEC + Cl‐amidine vs NEC) and the histological intestinal injury scoring did not differ between NEC and NEC + Cl‐amidine groups (Figure 4B,C). H&E examination of the ileal section in the sham group showed healthy villi and submucosal structure. In contrast, sections from both the NEC and the NEC + Cl‐amidine groups showed moderate‐to‐severe injury with mid‐villous damage.

As NET formation is an integral component of innate immunity via bacterial clearance,^34^ we postulated that NET inhibition in the model results in increased bacterial translocation. We, therefore, set up a separate experiment where mice pups’ blood culture was collected after *Klebsiella* gavage. First, we examined the bacterial load in mouse peripheral blood in the NEC group at 1‐, 3‐, 6‐, and 10‐hours post‐bacterial gavage. Notably, bacteraemia was significantly higher at 1 hour post‐gavage, followed by the 3‐hours time point and completely cleared from the peripheral blood at the 6‐ and 10‐hours post‐gavage time points (Supplemental Figure 3A). This was in parallel with the elevated nucleosome levels, specifically at the 1‐ and 3‐hours time point (Supplemental Figure 3B). Next, we compared the CFU/mL in the blood at the 1‐hour time point in both the NEC and NEC + Cl‐amidine group. Bacteraemia was significantly higher in the NEC + Cl‐amidine pups than in NEC alone (Figure 4D; *P* = 0.0074). These data suggest that Cl‐amidine‐associated NET inhibition appears to lead to a defect in bacterial clearance, eventually leading to bacteraemia and significantly increased mortality in NEC pups.

### 3.4 Effects of NET inhibition on leucocytes, platelets, systemic inflammation and organ function

**FIGURE 5:**
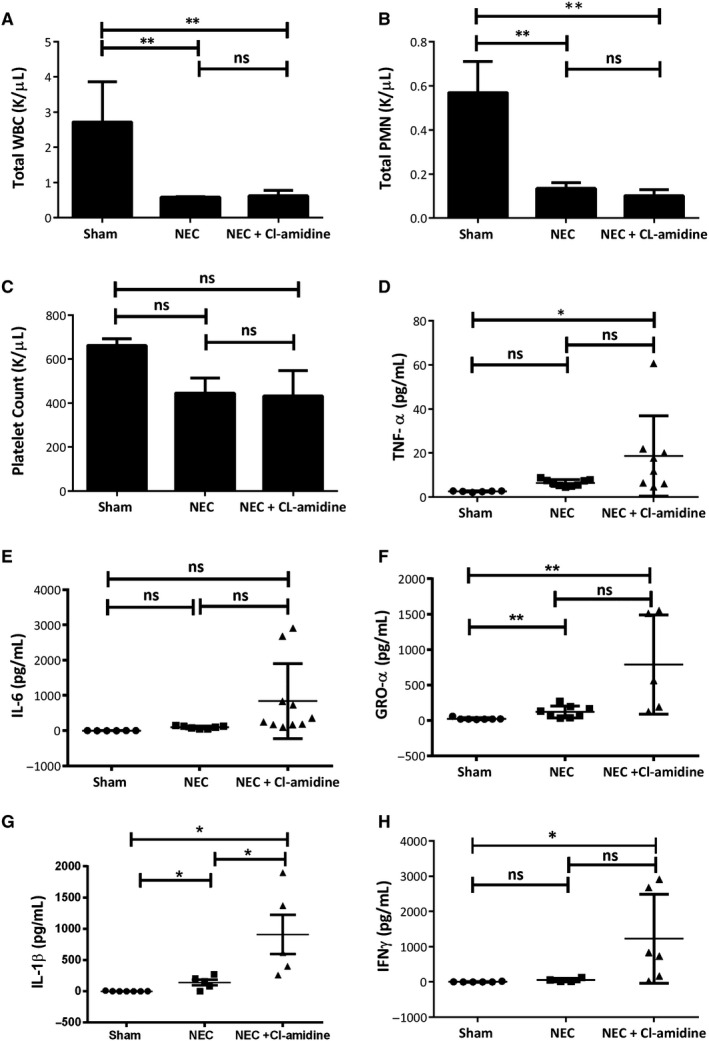
Leucocyte (A, total white blood cells [WBC], and B, neutrophils) and platelet (C) numbers in mouse pups subjected to NEC. Biological replicates, n ≥ 4. Values denote mean ± SEM by one‐way ANOVA **P < 0.01, ns*,* *P* > .05. D to H, Plasma cytokine levels in mouse pups subjected to NEC. Biological replicates, n ≥ 6. Values denote mean ± SEM by one‐way ANOVA. *P < 0.05, **P < 0.01, ns*,* *P* > 0.05. IL, interleukin; IFNγ, interferon gamma; K/µL, thousands per cubic millilitre; GRO‐α, growth‐regulated oncogene; NEC, necrotizing enterocolitis; PMN, polymorphonuclear neutrophils; TNF, tumour necrosis factor; WBC, white blood cells

To further characterize the effects of Cl‐amidine treatment and NET inhibition on the DK‐induced NEC model, we investigated changes in leucocytes, platelets, organ function and cytokine levels. As expected, total white blood cells (WBC) and neutrophils decreased significantly in pups subjected to NEC compared with shams, whereas platelets trended slightly lower in NEC pups (Figure 5A‐C; *P* = 0.0037 for WBC; *P* = 0.009 for neutrophils; *P* = 0.2643 for platelets). Levels of WBC, neutrophils and platelets did not differ significantly between NEC and NEC + Cl‐amidine pups (Figure 5A‐C). The significant reduction in WBC and neutrophil counts, and trend towards lower platelets among NEC pups likely indicates a massive infiltration of these cells from the systemic circulation into affected tissues.^35^ These data also indicate that, whereas Cl‐amidine treatment may have altered the functionality of neutrophils, in particular, the total quantities or percentages of these blood cells were not altered by NET inhibition.

As a disease characterized by hyperinflammation, NEC is commonly associated with elevated proinflammatory cytokine levels. As most mouse pups do not survive the NEC challenge, we measured systemic cytokines at 3‐hours time point after bacterial gavage. Mous pups in the NEC group had a slight but not statistically significant increase in some of the proinflammatory cytokines, likely because of the timing of collection. In mouse pups subjected to the DK NEC model, Cl‐amidine treatment induced a shift of the cytokine profile towards increased inflammation (Figure 5D‐H). Levels of TNF, IL‐6, GRO‐α, IL‐1β and IFNγ trended upwards in NEC pups compared with shams and, with the exception of IL‐6, were significantly increased in NEC + Cl‐amidine pups compared with shams (*P* = 0.0317 for TNF; *P* = 0.0333 for GRO‐α; *P* = 0.0029 for IL‐1β; *P* = 0.0442 for IFNγ). Additionally, IL‐1β levels were significantly elevated in NEC + Cl‐amidine compared with NEC pups (Figure 5G; *P* = 0.0161). Furthermore, NET inhibition in the model was associated with increased levels of blood urea nitrogen (BUN), creatinine and alanine transaminase (ALT) (Supplemental Figure 4A‐C). NET inhibition appears to further systemic inflammation and organ injury in murine NEC, likely because of increased bacteraemia.

## 4 DISCUSSION

In this study, we demonstrate that neutrophil activation and NET formation occur in both human and mouse NEC tissues. These data, and those of others,^10^, ^11^, ^12^ indicate that NETs, and by association circulating nucleosomes,^24^, ^36^ potentially play a role in the pathogenesis of NEC, given their enhanced presence during both human and mouse disease.

Neutrophils are a critical component of the innate immune system. These cells eradicate a variety of pathogens through reactive oxygen species bursts, degranulated antimicrobial peptides and enzymes, and the formation and release of NETs.^9^ During intestinal inflammation, and NEC in particular, neutrophils play an important role in the disease process. Following a disturbance in intestinal homeostasis, neutrophils are rapidly recruited from the blood to the lamina propria via chemokine production.^9^ Once present in the intestine, neutrophils attack translocating pathogens, often resulting in further tissue inflammation and damage.^37^ However, despite the collateral damage enacted by neutrophils, these cells appear to be so critical to the innate defence, and later for the resolution of tissue damage,^21^ that their eradication often leads to worsened outcome. In mice infected with *Staphylococcus aureus*, neutrophil depletion leads to decreased bacterial clearance, increased cytokine release and lower survival.^38^ Neutrophil eradication in rats and mice subjected to colitis results in increased intestinal bacterial translocation in the intestine and further disease progression.^39^ In murine NEC, depletion of neutrophils worsens the intestinal injury via decreased bacterial clearance, resulting in significantly decreased survival.^5^ Finally, preterm human infants with early‐onset neutropenia are at increased risk of developing NEC.^40^ Rather than deplete neutrophils entirely, we sought to determine the effects of inhibiting just one of the proinflammatory components of neutrophil defence, namely the production of NETs. However, data implicating the degree to which NETs either inhibit or exacerbate the disease progression are controversial. In the caecal ligation and puncture (CLP) mouse model of abdominal sepsis, NET formation drives pulmonary neutrophil infiltration and tissue injury, largely through an up‐regulation of inflammatory cytokines. In a dual‐hit model of haemorrhagic shock and sepsis in mice, NET formation in wild‐type mice is associated with increased end‐organ damage and mortality compared to mice with a deficiency in NET production.^41^ Mice with deficient NET production subjected to CLP sepsis are partially protected from shock, but do not succumb to increased levels of bacteraemia, suggesting the formation of NETs may significantly drive disease progression in sepsis.^42^ On the other hand, NETs proved indispensable in preventing systemic infection by circulating bacteria during severe murine sepsis, despite the complication of collateral hepatic tissue damage.^43^ NET depletion via DNAse administration in a mouse CLP model significantly increased susceptibility to polymicrobial sepsis because of enhanced systemic bacterial dissemination.^34^

Cl‐amidine, a pan‐PAD inhibitor, effectively inhibits NET formation in neutrophils, both in vitro and in vivo.^44^ PAD inhibition through Cl‐amidine treatment ameliorates endothelial, kidney and epithelial damage in mice suffering from lupus.^44^ In a mouse model of colitis, clinical symptoms of the disease were reduced with both prophylactic and post‐onset treatment with Cl‐amidine, potentially driven by increased apoptosis of inflammatory leucocytes and epithelial cells.^28^ When administered in a mouse model of CLP‐induced sepsis, Cl‐amidine treatment prevented mortality^27^ through a broad range of effects on systemic immunity, including reduced proinflammatory cytokine release.^45^ Finally, in an LPS/formula‐feeding/hypoxia mouse model of NEC, Cl‐amidine treatment reduced tissue inflammation and damage and increased survival.^10^

We aimed to elucidate the effects of inhibiting the production of NETs in the presence of bacteraemia during disease progression by administering Cl‐amidine in a different murine model of NEC, the DK model.^26^ We found that plasma nucleosomes in pups treated with Cl‐amidine trend towards lower levels, but not significantly, which indicates that circulating nucleosomes may originate from non‐NET‐related sources, such as tissue injury.^42^ Whereas treatment with Cl‐amidine in the DK mouse model of NEC has no significant effect on circulating neutrophils, platelets or total WBC count in comparison to NEC pups, NET inhibition resulted in systemic inflammation through an up‐regulation of proinflammatory cytokine production. Importantly, inhibition of NET formation did not lower intestinal injury scores, but resulted in bacteraemia and significantly hastened mortality compared with NEC pups. Altogether, our data indicate that the formation of NETs during NEC reduces intestinal bacterial translocation and lessens systemic inflammation in the DK mouse model. We suggest that the reduction of NETs in this setting of murine experimental NEC could worsen the consequences of systemic bacterial propagation and lead to rapid disease progression and enhanced mortality.

The formation of NETs may represent the epitome of a physiological trade‐off. This is particularly evident in sepsis, a highly complex disease consisting of both hyperinflammatory and compensatory anti‐inflammatory periods.^18^ Studies in sepsis, thus far, indicate that NET formation may be integral and protective in early stages in the prevention of systemic bacterial infection,^34^ but potentially overly proinflammatory and a significant cause of thrombosis, organ damage and secondary infection in later stages of the disease.^46^ We believe, based on our data, that NETs in NEC may dictate a very similar pattern: NEC‐induced NETs may be pivotal in early stages of the disease in preventing bacteraemia, but potentially detrimental in later stages when the scale has tipped towards massive tissue destruction through the accumulation of hyperinflammatory processes. In the DK mouse model of NEC, our data demonstrate that NET‐producing neutrophils are critical to the clearance of bacteria. Additionally, the loss of NET functionality appears to lead to increased systemic inflammation via up‐regulated proinflammatory cytokine production, eventually leading to bacteraemia and significantly decreased survival. This is consistent with the study reported by Saha et al,^47^ where NET inhibition, genetically or chemically using Cl‐amidine, in *Citrobacter rodentium* intestinal infection model, was associated with worsening pathology, decreased bacterial clearance, increased inflammation and increased dissemination. Taken together, our findings highlight the critical role of NETs in protecting intestinal pathologies associated with bacterial infection.

Our study is subject to several limitations. Because of the multi‐factorial nature of NEC, a variety of animal models are utilized to model NEC pathogenesis, each focusing on a unique aspect or suite of factors thought to contribute strongly to the human disease.^48^ Despite their differences, they all result in the characteristic histopathological features seen in human NEC: mucosal oedema, epithelial sloughing and villous atrophy.^49^ We studied NET inhibition utilizing the DK model of murine NEC to determine its effects on bacteria‐induced intestinal damage. This model utilizes disruption of the physiologically relevant Paneth cells followed by enteral gavage of live *Klebsiella pneumoniae* in 14‐day‐old mice.^26^ This model offers the advantage of timing that most closely mimics the human intestinal tract,^50^ and is highly relevant as infants who develop NEC have significantly lower Paneth cells and a similar bacterial dysbiosis pattern before NEC development.^33^, ^51^, ^52^, ^53^ Whereas no single organism is causative of NEC, studies suggest that alterations of the intestinal microbiota are either directly responsible or are associated with NEC development.^53^ Infants who develop NEC often have positive bacterial cultures from their peripheral blood or peritoneum.^54^ Also, the classic radiologic sign of pneumatosis intestinalis (intramural gas found in the intestinal wall) is suggested to occur after bacterial translocation across the epithelial barrier.^54^ Others^10^ have studied aspects of NET formations in a murine NEC model dependent upon formula feeding, LPS administration and periods of hypoxia. The strikingly different outcome of NET inhibition in these studies is likely derived from the use of distinct animal models, each contributing significant differences in time to disease development, age and developmental status of the animals, physiological mechanism(s) of disease development, live (and replicating) bacteria compared with static and Gram‐negative bacterial cell wall components. Whereas it is accepted that NETs play a role in the disease process, future studies using additional animal models are still needed to reach a consensus on important aspects of NET release and NET inhibition in the setting of NEC.

Additionally, the circulating nucleosomes identified and measured in our studies may represent a pool of histone‐DNA complexes contributed from a variety of sources, which could include both NETs^17^ and cells dying of other means.^25^, ^42^ Circulating nucleosome levels were significantly increased in both human neonatal and mouse NEC samples compared with controls, in accordance with both the analogous disease sepsis, denoting increased nucleosome levels^55^, ^56^ and increased cell‐free DNA described for NEC.^10^, ^12^ Regardless of the source, circulating nucleosomes potentially involved in the pathogenesis of NEC could represent a valuable biomarker of inflammation and infection.^57^

It is known that mice exhibit a smaller percentage of leucocytes as neutrophils compared to humans.^58^ Mouse neutrophils are further hampered by the inability to produce defensins, a strategy commonly employed by human neutrophils.^59^ Despite these potential hurdles, Cl‐amidine is effective in inhibiting NET formation by neutrophils.^10^, ^60^ This information together with the profound effect that Cl‐amidine treatment had on bacteraemia in our DK NEC mouse model and led us to believe that the role of neutrophil‐derived NETs in the innate defence may be even more important than previously realized. Further hindering the functionality of an already comparatively small percentage of neutrophils, lacking in the ability to produce defensins, may explain the phenotype induced by Cl‐amidine treatment in the DK mouse model of NEC. As human neonatal neutrophils are also characterized by reduced functionality, eliminating a critical component of the innate immune defence, namely NET formation, may prove to have dire consequences.

In conclusion, our study highlights the importance of neutrophil‐mediated NET formation in the protection against systemic bacterial dissemination during NEC. NEC mortality was increased following NET inhibition via Cl‐amidine administration, likely through bacteraemia. The effects of NET formation may be disease‐ and model‐specific, and in NEC, they depend largely upon the level of intestinal bacterial translocation. NETs appear to play an integral role in innate defence, and given the already fragile state of the preterm neonatal immune system, hindering a primary mechanism of bacterial clearance may not represent an effective approach to combating the disease.

## CONFLICT OF INTEREST

The authors declare no competing conflict of interest.

## AUTHOR CONTRIBUTIONS

HC, KB and JE performed the animal experiment; HC, RSK, KB and JE performed assays; HC, RSK, RS, KB, JE and FL analysed the data; HC, CL and FL designed the experiments, KB and HC wrote the manuscript; CL, ME, MC and BW provided critical revision of the manuscript. All authors read and approved the manuscript.

## Supporting information

Fig S1‐S4

## Data Availability

The data generated or analysed during this study are available from the corresponding author on reasonable request.

## References

[1] Neu J , Walker WA . Necrotizing enterocolitis. New Engl J Med. 2011;364:255‐264. 21247316 10.1056/NEJMra1005408 PMC3628622

[2] Patel RM , Kandefer S , Walsh MC , et al. Causes and timing of death in extremely premature infants from 2000 through 2011. New Engl J Med. 2015;372:331‐340. 25607427 10.1056/NEJMoa1403489 PMC4349362

[3] Hull MA , Fisher JG , Gutierrez IM , et al. Mortality and management of surgical necrotizing enterocolitis in very low birth weight neonates: a prospective cohort study. J Am Coll Surg. 2014;218:1148‐1155. 24468227 10.1016/j.jamcollsurg.2013.11.015

[4] Claud EC , Walker WA . Bacterial colonization, probiotics, and necrotizing enterocolitis. J Clin Gastroenterol. 2008;42(Suppl 2):S46‐S52. 18520617 10.1097/MCG.0b013e31815a57a8

[5] Emami CN , Mittal R , Wang L , et al. Role of neutrophils and macrophages in the pathogenesis of necrotizing enterocolitis caused by *Cronobacter sakazakii* . J Surg Res. 2012;172:18‐28. 21601887 10.1016/j.jss.2011.04.019 PMC3169739

[6] Stefanutti G , Lister P , Smith VV , et al. P‐selectin expression, neutrophil infiltration, and histologic injury in neonates with necrotizing enterocolitis. J Pediatr Surg. 2005;40:942‐7; discussion 7‐8. 15991175 10.1016/j.jpedsurg.2005.03.027

[7] Musemeche C , Caplan M , Hsueh W , et al. Experimental necrotizing enterocolitis: the role of polymorphonuclear neutrophils. J Pediatr Surg. 1991;26:1047‐1050; discussion 9‐50. 1941482 10.1016/0022-3468(91)90671-f

[8] Papayannopoulos V . Neutrophil extracellular traps in immunity and disease. Nat Rev Immunol. 2018;18:134‐147. 28990587 10.1038/nri.2017.105

[9] Fournier BM , Parkos CA . The role of neutrophils during intestinal inflammation. Mucosal Immunol. 2012;5:354‐366. 22491176 10.1038/mi.2012.24

[10] Vincent D , Klinke M , Eschenburg G , et al. NEC is likely a NETs dependent process and markers of NETosis are predictive of NEC in mice and humans. Sci Rep. 2018;8:12612. 30135601 10.1038/s41598-018-31087-0 PMC6105661

[11] MacQueen BC , Christensen RD , Yost CC , et al. Elevated fecal calprotectin levels during necrotizing enterocolitis are associated with activated neutrophils extruding neutrophil extracellular traps. J Perinatol. 2016;36:862‐869. 27388941 10.1038/jp.2016.105 PMC5045760

[12] Klinke M , Vincent D , Trochimiuk M , et al. Degradation of extracellular DNA significantly ameliorates necrotizing enterocolitis severity in mice. J Surg Res. 2019;235:513‐520. 30691836 10.1016/j.jss.2018.10.041

[13] Brinkmann V . Neutrophil extracellular traps in the second decade. J Innate Immun. 2018;10:414‐421. 29909412 10.1159/000489829 PMC6784051

[14] Wang Y , Li M , Stadler S , et al. Histone hypercitrullination mediates chromatin decondensation and neutrophil extracellular trap formation. J Cell Biol. 2009;184:205‐213. 19153223 10.1083/jcb.200806072 PMC2654299

[15] Farrera C , Fadeel B . Macrophage clearance of neutrophil extracellular traps is a silent process. J Immunol. 1950;2013(191):2647‐2656. 10.4049/jimmunol.1300436 23904163

[16] Yost CC , Cody MJ , Harris ES , et al. Impaired neutrophil extracellular trap (NET) formation: a novel innate immune deficiency of human neonates. Blood. 2009;113:6419‐6427. 19221037 10.1182/blood-2008-07-171629 PMC2710935

[17] Brill A , Fuchs TA , Savchenko AS , et al. Neutrophil extracellular traps promote deep vein thrombosis in mice. J Thromb Haemost. 2012;10:136‐144. 22044575 10.1111/j.1538-7836.2011.04544.x PMC3319651

[18] Camicia G , Pozner R , de Larranaga G . Neutrophil extracellular traps in sepsis. Shock. 2014;42:286‐294. 25004062 10.1097/SHK.0000000000000221

[19] Caudrillier A , Kessenbrock K , Gilliss BM , et al. Platelets induce neutrophil extracellular traps in transfusion‐related acute lung injury. J Clin Invest. 2012;122:2661‐2671. 22684106 10.1172/JCI61303 PMC3386815

[20] Marcos V , Nussbaum C , Vitkov L , et al. Delayed but functional neutrophil extracellular trap formation in neonates. Blood. 2009;114(23):4908‐4911; author reply 11–2. 19965699 10.1182/blood-2009-09-242388

[21] Denning TL , Bhatia AM , Kane AF , et al. Pathogenesis of NEC: role of the innate and adaptive immune response. Semin Perinatol. 2017;41:15‐28. 27940091 10.1053/j.semperi.2016.09.014 PMC5484641

[22] Kusunoki Y , Nakazawa D , Shida H , et al. Peptidylarginine deiminase inhibitor suppresses neutrophil extracellular trap formation and MPO‐ANCA production. Front Immunol. 2016;7:227. 27375623 10.3389/fimmu.2016.00227 PMC4896908

[23] Walsh MC , Kliegman RM . Necrotizing enterocolitis: treatment based on staging criteria. Pediatr Clin North Am. 1986;33:179‐201. 3081865 10.1016/S0031-3955(16)34975-6 PMC7131118

[24] Kim J‐E , Lee N , Gu J‐Y , et al. Circulating levels of DNA‐histone complex and dsDNA are independent prognostic factors of disseminated intravascular coagulation. Thromb Res. 2015;135:1064‐1069. 25843168 10.1016/j.thromres.2015.03.014

[25] Marsman G , Zeerleder S , Luken BM . Extracellular histones, cell‐free DNA, or nucleosomes: differences in immunostimulation. Cell Death Dis. 2016;7:e2518. 27929534 10.1038/cddis.2016.410 PMC5261016

[26] Zhang C , Sherman MP , Prince LS , et al. Paneth cell ablation in the presence of *Klebsiella pneumoniae* induces necrotizing enterocolitis (NEC)‐like injury in the small intestine of immature mice. Dis Mod Mech. 2012;5:522‐532. 10.1242/dmm.009001 PMC3380715 22328592

[27] Li Y , Liu Z , Liu B , et al. Citrullinated histone H3: a novel target for the treatment of sepsis. Surgery. 2014;156:229‐234. 24957671 10.1016/j.surg.2014.04.009 PMC4267527

[28] Chumanevich AA , Causey CP , Knuckley BA , et al. Suppression of colitis in mice by Cl‐amidine: a novel peptidylarginine deiminase (PAD) inhibitor. Am J Physiol Gastrointest Liver Physiol. 2011;300:G929‐G938. 21415415 10.1152/ajpgi.00435.2010 PMC3119113

[29] Jilling T , Lu J , Jackson M , et al. Intestinal epithelial apoptosis initiates gross bowel necrosis in an experimental rat model of neonatal necrotizing enterocolitis. Pediatr Res. 2004;55:622‐629. 14764921 10.1203/01.PDR.0000113463.70435.74

[30] Rao RM , Betz TV , Lamont DJ , et al. Elastase release by transmigrating neutrophils deactivates endothelial‐bound SDF‐1alpha and attenuates subsequent T lymphocyte transendothelial migration. J Exp Med. 2004;200:713‐724. 15381727 10.1084/jem.20040499 PMC2211969

[31] Hemmers S , Teijaro JR , Arandjelovic S , et al. PAD4‐mediated neutrophil extracellular trap formation is not required for immunity against influenza infection. PLoS ONE. 2011;6:e22043. 21779371 10.1371/journal.pone.0022043 PMC3133614

[32] Chaaban H , Keshari RS , Silasi‐Mansat R , et al. Inter‐alpha inhibitor protein and its associated glycosaminoglycans protect against histone‐induced injury. Blood. 2015;125:2286‐2296. 25631771 10.1182/blood-2014-06-582759 PMC4383802

[33] Lueschow SR , Stumphy J , Gong H , et al. Loss of murine Paneth cell function alters the immature intestinal microbiome and mimics changes seen in neonatal necrotizing enterocolitis. PLoS ONE. 2018;13:e0204967. 30273395 10.1371/journal.pone.0204967 PMC6166990

[34] Meng W , Paunel‐Görgülü A , Flohé S , et al. Depletion of neutrophil extracellular traps in vivo results in hypersusceptibility to polymicrobial sepsis in mice. Crit Care. 2012;16:R137. 22835277 10.1186/cc11442 PMC3580722

[35] Hassan M , Yasmeen BHN . Neutropenia in neonate—an overview. North Int Med College J. 2016;7:149‐152.

[36] Thomas GM , Carbo C , Curtis BR , et al. Extracellular DNA traps are associated with the pathogenesis of TRALI in humans and mice. Blood. 2012;119:6335‐6343. 22596262 10.1182/blood-2012-01-405183 PMC3383196

[37] Cooper PR , Palmer LJ , Chapple ILC . Neutrophil extracellular traps as a new paradigm in innate immunity: friend or foe? Periodont. 2000;2013(63):165‐197. 10.1111/prd.12025 23931060

[38] Robertson CM , Perrone EE , McConnell KW , et al. Neutrophil depletion causes a fatal defect in murine pulmonary *Staphylococcus aureus* clearance. J Surg Res. 2008;150:278‐285. 18621398 10.1016/j.jss.2008.02.009 PMC2605623

[39] Kühl AA , Kakirman H , Janotta M , et al. Aggravation of different types of experimental colitis by depletion or adhesion blockade of neutrophils. Gastroenterology. 2007;133:1882‐1892. 18054560 10.1053/j.gastro.2007.08.073

[40] Christensen RD , Yoder BA , Baer VL , et al. Early‐onset neutropenia in small‐for‐gestational‐age infants. Pediatrics. 2015;136:e1259‐e1267. 26459642 10.1542/peds.2015-1638

[41] Biron BM , Chung C‐S , Chen Y , et al. PAD4 deficiency leads to decreased organ dysfunction and improved survival in a dual insult model of hemorrhagic shock and sepsis. J Immunol. 2018;200:1817‐1828. 29374076 10.4049/jimmunol.1700639 PMC5821587

[42] Martinod K , Fuchs TA , Zitomersky NL , et al. PAD4‐deficiency does not affect bacteremia in polymicrobial sepsis and ameliorates endotoxemic shock. Blood. 2015;125:1948‐1956. 25624317 10.1182/blood-2014-07-587709 PMC4366625

[43] McDonald B , Urrutia R , Yipp B , et al. Intravascular neutrophil extracellular traps capture bacteria from the bloodstream during sepsis. Cell Host Microbe. 2012;12:324‐333. 22980329 10.1016/j.chom.2012.06.011

[44] Knight JS , Subramanian V , O'Dell AA , et al. Peptidylarginine deiminase inhibition disrupts NET formation and protects against kidney, skin and vascular disease in lupus‐prone MRL/lpr mice. Ann Rheum Dis. 2015;74:2199‐2206. 25104775 10.1136/annrheumdis-2014-205365 PMC4320672

[45] Zhao T , Pan B , Alam HB , et al. Protective effect of Cl‐amidine against CLP‐induced lethal septic shock in mice. Sci Rep. 2016;6:36696. 27819302 10.1038/srep36696 PMC5098180

[46] Kambas K , Mitroulis I , Apostolidou E , et al. Autophagy mediates the delivery of thrombogenic tissue factor to neutrophil extracellular traps in human sepsis. PLoS ONE. 2012;7:e45427. 23029002 10.1371/journal.pone.0045427 PMC3446899

[47] Saha P , Yeoh BS , Xiao X , et al. PAD4‐dependent NETs generation are indispensable for intestinal clearance of *Citrobacter rodentium* . Mucosal Immunol. 2019;12:761‐771. 30710097 10.1038/s41385-019-0139-3 PMC6519124

[48] Sulistyo A , Rahman A , Biouss G , et al. Animal models of necrotizing enterocolitis: review of the literature and state of the art. Innov Surg Sci. 2018;3:87‐92. 31579771 10.1515/iss-2017-0050 PMC6604570

[49] Nolan LS , Wynn JL , Good M . Exploring clinically‐relevant experimental models of neonatal shock and necrotizing enterocolitis. Shock. 2020;53(5):596‐604. 31977960 10.1097/SHK.0000000000001507 PMC7376956

[50] Stanford AH , Gong H , Noonan M , et al. A direct comparison of mouse and human intestinal development using epithelial gene expression patterns. Pediatr Res. 2019. 10.1038/s41390-019-0472-y. [Epub ahead of print]. PMC6930976 31242501

[51] Pammi M , Cope J , Tarr PI , et al. Intestinal dysbiosis in preterm infants preceding necrotizing enterocolitis: a systematic review and meta‐analysis. Microbiome. 2017;5:31. 28274256 10.1186/s40168-017-0248-8 PMC5343300

[52] Underwood MA . Paneth cells and necrotizing enterocolitis. Gut Microbes. 2012;3:562‐565. 22895084 10.4161/gmic.21738 PMC3495794

[53] Neu J , Pammi M . Necrotizing enterocolitis: the intestinal microbiome, metabolome and inflammatory mediators. Semin Fetal Neonatal Med. 2018;23:400‐405. 30172660 10.1016/j.siny.2018.08.001

[54] Coggins SA , Wynn JL , Weitkamp J‐H . Infectious causes of necrotizing enterocolitis. Clin Perinatol. 2015;42:133‐154. 25678001 10.1016/j.clp.2014.10.012 PMC4328138

[55] Zeerleder S , Zwart B , Wuillemin WA , et al. Elevated nucleosome levels in systemic inflammation and sepsis. Crit Care Med. 2003;31:1947‐1951. 12847387 10.1097/01.CCM.0000074719.40109.95

[56] Chen QiXing , Ye L , Jin YuHong , et al. Circulating nucleosomes as a predictor of sepsis and organ dysfunction in critically ill patients. Int J Infect Dis. 2012;16:e558‐e564. 22609014 10.1016/j.ijid.2012.03.007

[57] Patel P , Walborn A , Rondina M , et al. Markers of inflammation and infection in sepsis and disseminated intravascular coagulation. Clin Appl Thromb Hemost. 2019;25. 10.1177/1076029619843338. PMC6714897 30991817

[58] Mestas J , Hughes CC . Of mice and not men: differences between mouse and human immunology. J Immunol. 2004;172:2731‐2738. 14978070 10.4049/jimmunol.172.5.2731

[59] Ganz T . Defensins: antimicrobial peptides of innate immunity. Nat Rev Immunol. 2003;3:710‐720. 12949495 10.1038/nri1180

[60] Li P , Li M , Lindberg MR , et al. PAD4 is essential for antibacterial innate immunity mediated by neutrophil extracellular traps. J Exp Med. 2010;207:1853‐1862. 20733033 10.1084/jem.20100239 PMC2931169

