# Supplementary material for: Neutrophil extracellular trap inhibition increases inflammation, bacteraemia and mortality in murine necrotizing enterocolitis
Source: J Cell Mol Med. 2020 Jun 8;25(23):10814–24. doi: 10.1111/jcmm.15338 (PMC8642694; doi:10.1111/jcmm.15338)
Supplement: Supplementary file 1 — Fig S1‐S4 [file JCMM-25-10814-s001.docx]

**SUPPLEMENTAL INFORMATION**

**Neutrophil Extracellular Trap Inhibition Increases Inflammation, Bacteremia, and Mortality in Murine Necrotizing Enterocolitis**

Hala Chaaban, Kathryn Burge, Jeffrey Eckert, Ravi S. Keshari, Robert Silasi,

Cristina Lupu, Barbara Warner, Marilyn Escobedo, Micahel Caplan, and Florea Lupu

**
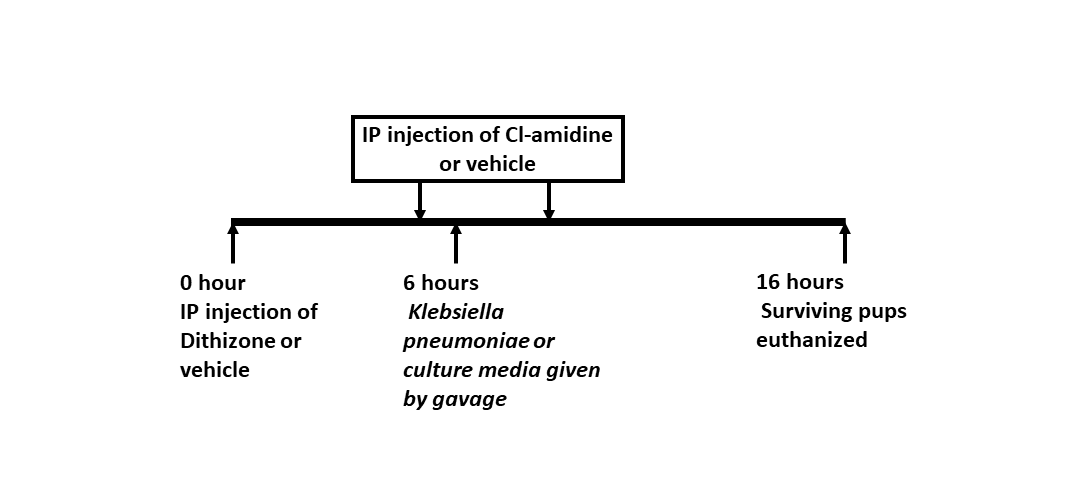
**

**Supplemental Fig. 1**: **Modified Paneth cell ablation and *Klebsiella* infection model**. Pups in the NEC groups received an *i.p*. injection of dithizone (33 mg/kg body-weight) or an equivalent volume of vehicle alone At six hours post-injection, pups received an enteral gavage of 1x10^8^ CFU /gram body-weight of *Klebsiella pneumoniae*. Pups in the Cl-amidine group received 40 mg/kg body-weight Cl-amidine 30 minutes before the bacterial challenge and 3 hours post-challenge. Pups were continuously monitored for distress and survival for 10 hours, after which surviving pups were euthanized, and blood and tissues were collected. NEC: necrotizing enterocolitis; CFU/ml: Colony-forming units/ml.

**
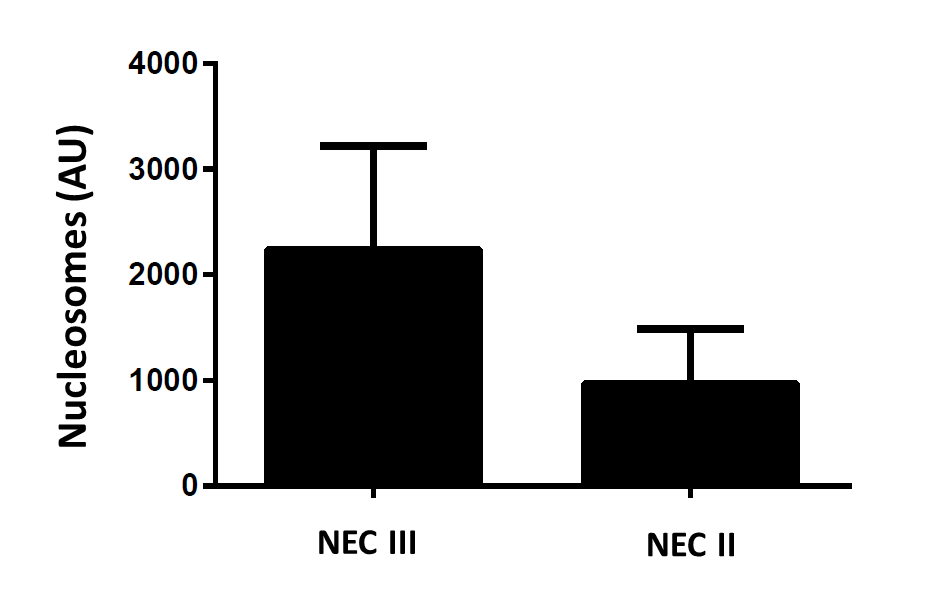
**

**Supplemental Fig. 2**: **Plasma nucleosome levels in preterm infants with NEC stage III compared to stage II.** Values denote mean ± SEM by student t-test. NEC: necrotizing enterocolitis; AU: absorbance units.

**
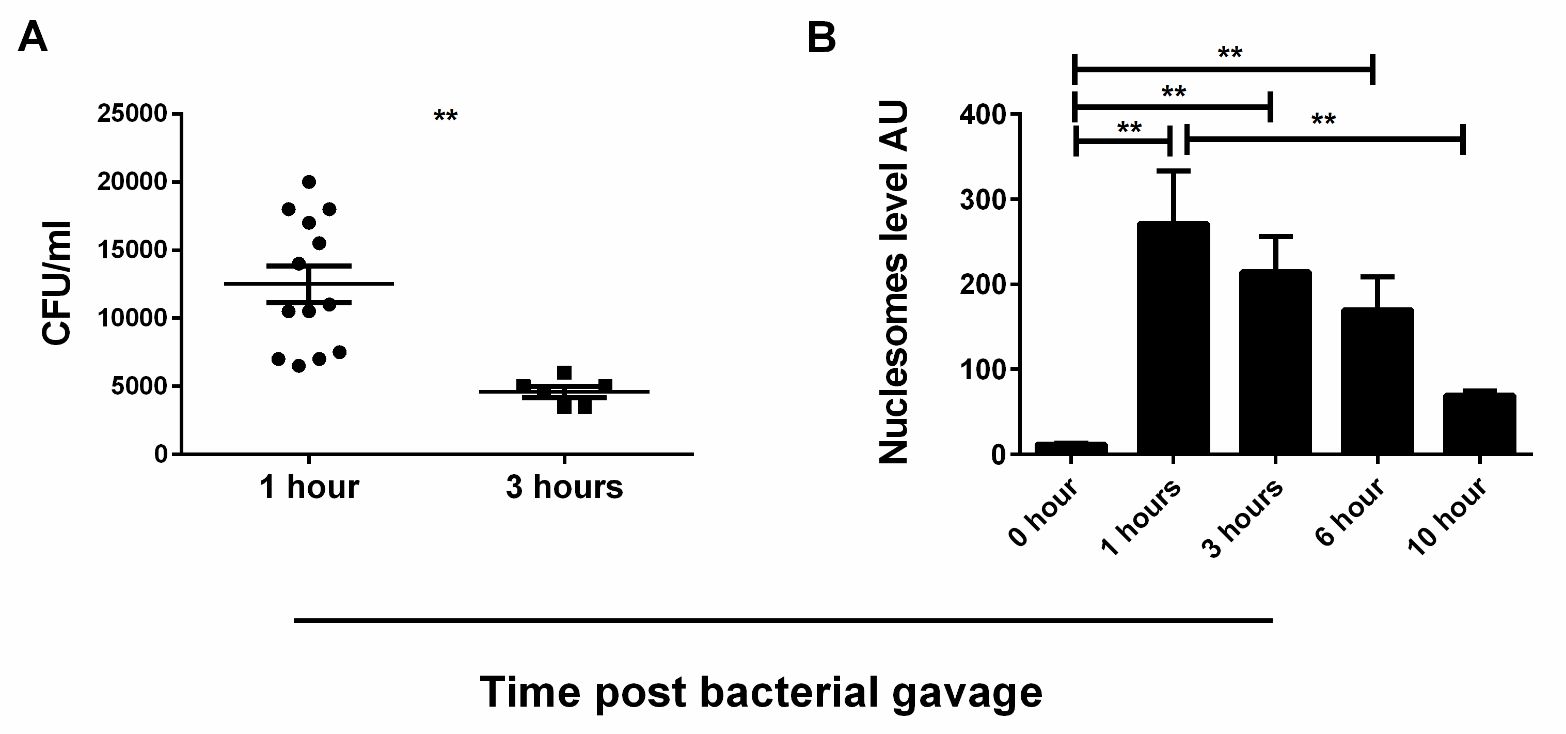
**

**Supplemental Fig. 3:** **A)** **Bacterial counts in mouse pups 1 and 3 -hours post- bacterial gavage**. ***p*<0.01. **B)** **Plasma nucleosome levels in mouse in the NEC group before, and at 1, 3, 6, and 10-hour post-gavage**. ***p*<0.01. Values denote mean ± SEM by student t-test or one-way ANOVA. CFU/ml: Colony-forming units/ml; AU: absorbance units.

**
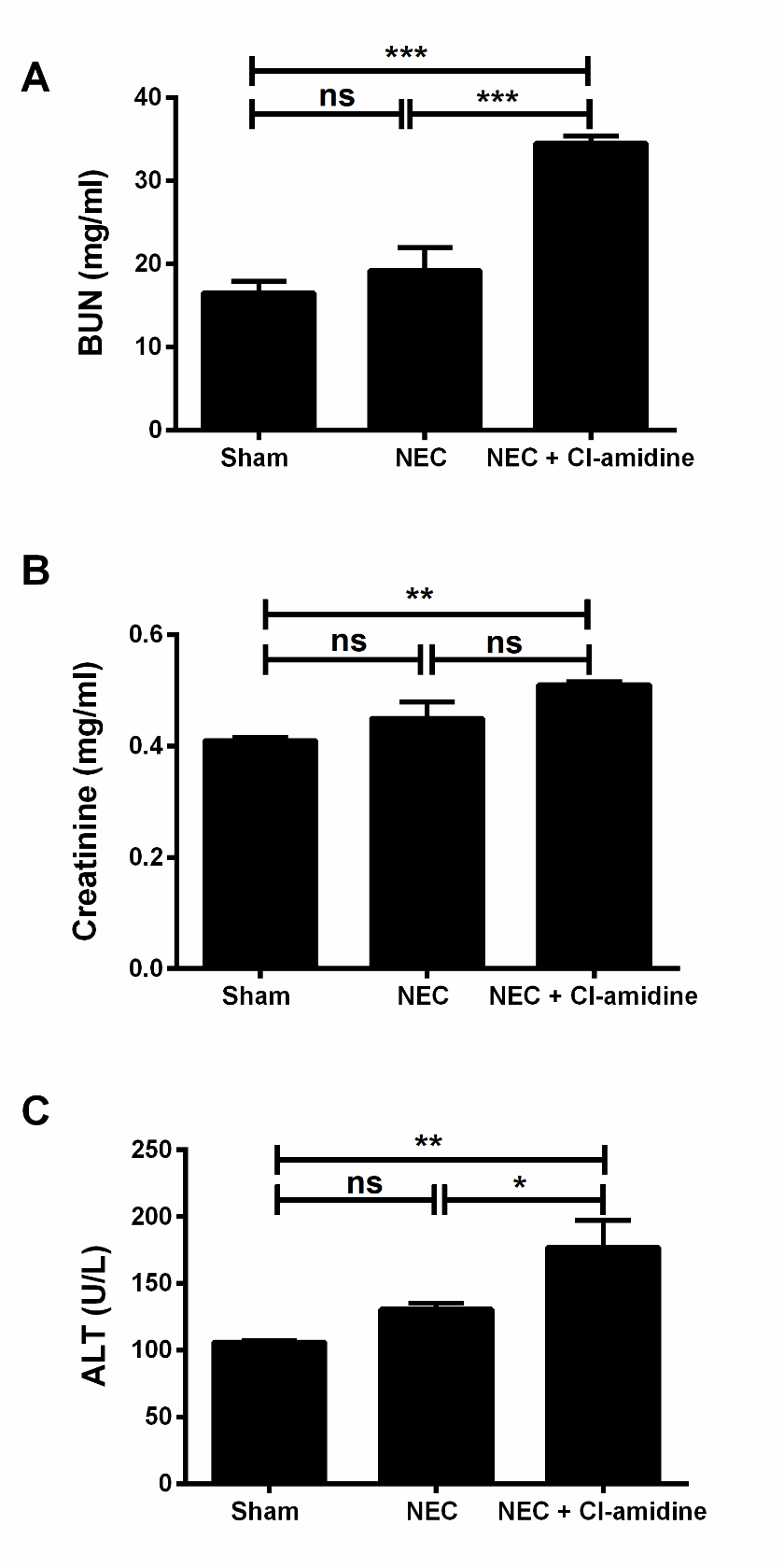
Supplemental Fig. 4:** **Plasma levels of BUN, Creatinine, and ALT in shams, NEC, and NEC + Cl amidine** using Catalyst Dx Chemistry Analyzer (IDEXX Laboratories, Inc.). Values denote mean ± SEM by one-way ANOVA. BUN: Blood urea nitrogen; ALT: Alanine aminotransferase.
